# An increased relative eosinophil count as a predictive dynamic biomarker in non‐small cell lung cancer patients treated with immune checkpoint inhibitors

**DOI:** 10.1111/1759-7714.15191

**Published:** 2023-12-12

**Authors:** Eiji Takeuchi, Hirokazu Ogino, Kensuke Kondo, Yoshio Okano, Seiya Ichihara, Michihiro Kunishige, Naoki Kadota, Hisanori Machida, Nobuo Hatakeyama, Keishi Naruse, Hiroshi Nokihara, Tsutomu Shinohara, Yasuhiko Nishioka

**Affiliations:** ^1^ Department of Clinical Investigation National Hospital Organization Kochi Hospital Kochi Japan; ^2^ Department of Respiratory Medicine and Rheumatology Graduate School of Biomedical Sciences, Tokushima University Tokushima Japan; ^3^ Department of Respiratory Medicine National Hospital Organization Kochi Hospital Kochi Japan; ^4^ Department of Pathology National Hospital Organization Kochi Hospital Kochi Japan; ^5^ Department of Community Medicine for Respirology Graduate School of Biomedical Sciences, Tokushima University Tokushima Japan

**Keywords:** dynamic biomarker, immune checkpoint inhibitors, increased eosinophil count, non‐small cell lung cancer, predictive factor

## Abstract

**Background:**

An increased relative eosinophil count (REC) has potential as a predictive biomarker for a beneficial clinical response and outcome to cancer immunotherapies. Therefore, the present study investigated the impact of an increased posttreatment REC on the prognosis of non‐small cell lung cancer (NSCLC) patients treated with immune checkpoint inhibitors (ICIs).

**Methods:**

We retrospectively reviewed all 151 patients diagnosed with NSCLC and treated with ICI monotherapy and blood test data between March 2016 and August 2021 at National Hospital Organization Kochi Hospital and Tokushima University.

**Results:**

A total of 151 patients with a mean age of 69 years were included. REC after 4 weeks of initial ICI monotherapy was higher than pretreatment REC in 87 patients but not in 64. REC after 4 weeks of the ICI treatment with and without an increased REC were 4.4 and 1.8%, respectively (*p* < 0.001). Disease control rates (DCR) were significantly higher in patients with than in those without an increased REC (84% vs. 47%, *p* < 0.001). The median overall survival (OS) of lung cancer patients with or without an increased REC were 674 and 234 days, respectively. A Kaplan–Meier univariate analysis revealed a significant difference in OS between the two groups (*p* < 0.001). A Cox proportional regression analysis identified an increased REC as an independent predictor of OS (*p* = 0.003).

**Conclusion:**

ICI‐treated NSCLC patients with an increased REC after 4 weeks of treatment had a better DCR and prognosis than the other patients examined.

## Graphical abstract

This is the first study using multivariate analysis to show that an increased REC after 4 weeks of treatment is an important predictive factor of lung cancer patients treated with ICIs. In daily clinical practice, an increased REC has potential as an early predictive dynamic biomarker of lung cancer patients treated with ICIs.

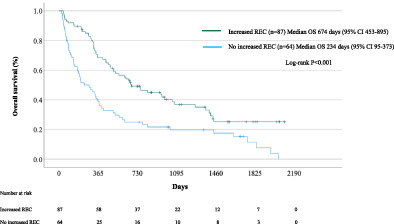

## INTRODUCTION

Immune checkpoint inhibitors (ICIs) have markedly changed the treatment of several malignancies, including lung cancer. However, not all patients benefit from ICIs. Several clinically relevant prognostic and predictive markers have been reported in non‐small cell lung cancer (NSCLC) patients treated with ICIs.^1^, ^2^ In clinical practice, programmed death ligand 1 (PD‐L1) is regarded as the most common biomarker to predict treatment responses.^3^, ^4^ However, the expression of PD‐L1 is dependent on the immunostaining of pathology specimens, and the biopsy site may not fully represent overall lung cancer.^4^ In addition, the expression of PD‐L1 may vary depending on where surgically resected specimens are stained and evaluated.^4^ Therefore, better biomarkers are needed in clinical practice.

Eosinophils are effector cells in allergic diseases and parasitic infections and have multiple functions that differ from those of neutrophils and lymphocytes.^5^, ^6^ Eosinophils have recently been suggested to regulate homeostatic processes at a steady state.^7^ Previous studies demonstrated that tumor‐associated eosinophils may prolong the survival of some cancer patients.^8^, ^9^, ^10^ We also reported that the prognosis of lung cancer patients with eosinophilic pleural effusion was better than that of patients with non‐eosinophilic pleural effusion.^11^ Overall survival (OS) was longer in ICI‐treated NSCLC patients with a pretreatment eosinophil count of 100 cells/μL or higher, but less than 500 cells/μL (100 ≤ Eo < 500) than in the other patients examined.^12^ Meta‐analyses revealed that tumor‐associated tissue eosinophils predicted favorable clinical outcomes in solid tumors.^13^ An increased relative eosinophil count (REC) at 3 weeks was identified as a significant predictive factor in urothelial carcinoma patients treated with pembrolizumab.^14^ In lung cancer patients, ICI‐treated NSCLC patients with eosinophilia during treatment had higher response rates (RR) and more prolonged treatment durations.^15^, ^16^, ^17^, ^18^

OS is the most robust indicator of cancer treatment outcomes. Nevertheless, the relationship between an increased REC and OS in lung cancer patients treated with ICIs has not yet been examined in detail.^19^ Therefore, we herein investigated the impact of an increased REC on the efficacy of ICIs for NSCLC and the prognosis of treated patients.

## METHODS

### Patients

We retrospectively reviewed all 151 patients diagnosed with advanced or metastatic NSCLC, treated with ICI monotherapy (nivolumab, pembrolizumab, and atezolizumab), and with available blood test data after 4 weeks between March 2016 and August 2021 at National Hospital Organization Kochi Hospital and Tokushima University.

### Data collection

We collected data on age, sex, smoking history, the Eastern Cooperative Oncology Group performance status (ECOG PS), baseline white blood cell count, neutrophil count, lymphocyte count, neutrophil‐to‐lymphocyte ratio in serum (sNLR), eosinophil count, C‐reactive protein (CRP), albumin (Alb), the histological type, PD‐L1 expression, the type of ICI, line of ICI, date of ICI initiation, and the status of death. We also collected data on white blood cell, neutrophil, lymphocyte, and eosinophil counts after approximately 2, 4, and 6 weeks of the initial ICI treatment. We examined data obtained on the primary lesion size (maximum diameter measured on chest computed tomography [CT]), the number of metastatic sites (a count of involved solid organs, not all sites), the status of specific metastasis (nonregional lymph nodes, the contralateral lung, pleura, brain, liver, kidney, adrenal gland, and bone), and stage (according to the eighth edition of the tumor‐node‐metastasis classification of lung cancer). CT was performed for a radiological evaluation before the ICI treatment. A radiographic complete response, partial response, stable disease, and progressive disease were defined according to the Response Evaluation Criteria in Solid Tumor, version 1.1.^20^ Objective RR and disease control rates (DCR) were calculated as a complete response plus a partial response and a complete response plus a partial response and stable disease, respectively. OS was calculated as the time from the start of ICI monotherapy to death from any cause.

### Statistical analysis

According to previous studies, median baseline CRP and Alb levels were selected as cutoff values,^21^ while 100 ≤ Eo < 500, sNLR < 5, and tumor size <5 cm were chosen as cutoff values.^12^, ^22^, ^23^ Categorical and continuous variables are summarized using descriptive statistics. The independent samples *t*‐test and paired *t*‐test were used to analyze differences between continuous variables. Pearson's chi‐squared test and Fisher's exact test were employed to analyze relationships between categorical variables. OS was evaluated as the period from when ICI was initiated to the day of death from any cause using the Kaplan–Meier method. A log‐rank test was performed to compare survival curves. A Cox proportional hazards model was used to estimate the hazard ratio (HR) with a 95% confidence interval (CI). Verification was performed with 1000 bootstrap resamples for internal validation. We conducted all statistical analyses using SPSS version 27.0 (IBM). *p*‐values are presented without adjustments for multiple comparisons in an exploratory manner.

## RESULTS

### Patient characteristics

The study included 151 advanced or metastatic NSCLC patients treated with ICI monotherapy. The clinical characteristics of enrolled patients are summarized in Table 1. The mean age of patients at the initiation of ICI therapy was 69 years; 117 (77%) were male, and 118 (78%) were ex‐ or current smokers. Most patients (90%) had an ECOG PS of 0–1. Twenty‐one patients (14%) had postoperative recurrence, 30 (20%) had stage III, and 100 (66%) had stage IV. Eighty‐nine patients (59%) exhibited an adenocarcinoma histology, while 43 (28%) showed a squamous cell carcinoma histology. PD‐L1 expression was ≥1% in 118 patients (78%) but was absent in 30 (20%). Forty patients (26%) received ICIs as first‐line therapy. A total of 111 patients (74%) received ICIs as a second‐line or later treatment. A total of 117 patients (77%) were treated with pembrolizumab, 33 (22%) with nivolumab, and one (1%) with atezolizumab. Thirteen patients (13%) had liver metastasis. Thirty‐four patients (23%) had brain metastasis. There were no patients with complications of parasitic infections or atopic or allergic diseases. Nine patients (6%) received oral steroids regularly during the treatment. The average REC was 2.7% before the ICI treatment and 3.3% after 4 weeks.

**TABLE 1:** Characteristics of the study population.

		Total	Increased REC	No increased REC	*p*‐value
		*n* = 151	*n* = 87	*n* = 64	
Age, years	Mean, (SD)	69 (9)	70 (9)	69 (9)	0.6^a^
Sex, n (%)	Male	117 (77)	67 (77)	50 (78)	1.0^b^
	Female	34 (23)	20 (23)	14 (22)	
Smoking history, n (%)	Yes	118 (78)	64 (74)	54 (84)	0.2^b^
	No	28 (19)	19 (22)	9 (14)	
ECOG PS, n (%)	0–1	136 (90)	80 (92)	56 (88)	0.4^b^
	2–4	15 (10)	7 (8)	8 (13)	
Stage, n (%)	Recurrence	21 (14)	12 (14)	9 (14)	0.8^c^
	III	30 (20)	19 (22)	11 (17)	
	IV	100 (66)	56 (64)	44 (69)	
Histological type, n (%)	Adeno	89 (59)	50 (57)	39 (61)	0.8^c^
	Squamous	43 (28)	27 (31)	16 (25)	
	Others	19 (13)	10 (11)	9 (14)	
PD‐L1, n (%)	<1%	30 (20)	13 (15)	17 (27)	0.06^c^
	≥1%	118 (78)	72 (83)	46 (72)	
	Missing	3 (2)	2 (2)	1 (2)	
Treatment line, n (%)	1	40 (26)	27 (31)	13 (20)	0.1^b^
	>1	111 (74)	60 (69)	51 (80)	
ICI drug, n (%)	Pembrolizumab	117 (77)	71 (82)	46 (72)	0.2^c^
	Nivolumab	33 (22)	16 (18)	17 (27)	
	Atezolizumab	1 (1)	0 (0)	1 (2)	
Liver metastasis, n (%)	No	138 (91)	80 (92)	58 (91)	0.7^c^
	Yes	13 (9)	7 (8)	6 (9)	
Brain metastasis, n (%)	No	117 (77)	64 (74)	53 (83)	0.2^c^
	Yes	34 (23)	23 (26)	11 (17)	
Steroids, n (%)	No	142 (94)	81 (93)	61 (95)	0.4^b^
	Yes	9 (6)	6 (7)	3 (5)	
Tumor size, mm	Mean (SD)	43 (23)	41 (22)	45 (25)	0.2^a^
White blood count/μL	Mean (SD)	6826 (2597)	6607 (2262)	7124 (2987)	0.2^a^
Neutrophils/μL	Mean (SD)	4647 (2231)	4509 (1983)	4833 (2535)	0.4^a^
Neutrophils, %	Mean (SD)	66 (9)	67 (9)	66 (9)	0.6^a^
Lymphocytes/μL	Mean (SD)	1440 (581)	1426 (533)	1458 (645)	0.7^a^
Lymphocytes, %	Mean (SD)	22 (9)	23 (8)	22 (9)	0.6 ^a^
Eosinophils/μL	Mean, (SD)	179 (187)	144 (126)	226 (239)	0.01^a^
Eosinophils, %	Mean (SD)	2.7 (2.5)	2.3 (2.0)	3.1 (3.9)	0.01^a^
sNLR, ratio	Mean (SD)	3.6 (2.0)	3.6 (1.9)	3.7 (2.1)	0.6^a^
CRP, mg/dL	Mean (SD)	2.3 (3.1)	2.0 (2.6)	2.8 (3.7)	0.1^a^
Alb, g/dL	Mean (SD)	3.5 (0.6)	3.6 (0.6)	3.4 (0.7)	0.02^a^
4w white blood count/μL	Mean (SD)	7929 (5170)	6709 (2633)	9588 (7023)	0.003^a^
4w neutrophils/μL	Mean (SD)	5625 (4993)	4408 (2455)	7278 (6806)	<0.001^a^
4w neutrophils, %	Mean (SD)	67 (12)	63 (11)	71 (13)	<0.001^a^
4w lymphocytes/μL	Mean (SD)	1516 (694)	1492 (617)	1549 (791)	0.6^a^
4w lymphocytes, %	Mean (SD)	22 (10)	24 (9)	20 (11)	0.01^a^
4w eosinophils/μL	Mean (SD)	218 (246)	280 (285)	134 (143)	<0.001^a^
4w eosinophils, %	Mean (SD)	3.3 (3.4)	4.4 (3.8)	1.8 (1.9)	<0.001^a^
4w sNLR, ratio	Mean (SD)	5.2 (7.5)	4.2 (6.4)	6.6 (8.6)	0.06^a^

Abbreviations: Alb, albumin; CRP, C‐reactive protein; ECOG PS, Eastern Cooperative Oncology Group performance Ssatus; ICI, immune checkpoint inhibitor; PD‐L1, programmed death ligand 1; REC, relative eosinophil count after 4 weeks of immune checkpoint inhibitor treatment; sNLR, neutrophil‐to‐lymphocyte ratio in serum; 4w, 4 weeks later.

^a^ Independent samples *t*‐test.

^b^ Fisher's exact test.

^c^ Chi‐squared test.

We examined changes over time in leukocyte counts based on the treatment effect (Table 2). Responders were patients with a complete or partial response, while non‐responders were those with stable or progressive disease. In the responder group, changes in neutrophil, lymphocyte, and eosinophil counts were examined after 4 and 6 weeks of initial ICI monotherapy. We analyzed data after 4 weeks when changes occurred earlier. Patients in the present study were divided into two groups: one with a higher REC than pretreatment REC after 4 weeks of initial ICI monotherapy and the other without a higher REC than pretreatment REC. REC before ICI therapy with and without an increased REC were 2.3 and 3.1%, respectively. A significant difference was observed between the two groups (*p* = 0.01). REC after 4 weeks of ICI therapy with and without an increased REC were 4.4 and 1.8%, respectively, with a significant difference (*p* < 0.001). The mean age of 87 patients with an increased REC was 70 years; 67 (77%) were male, and 64 (74%) were ex‐ or current smokers. Eighty patients (92%) had an ECOG PS of 0–1. Twelve patients (14%) had postoperative recurrence, 19 (22%) had stage III, and 56 (64%) had stage IV. Fifty patients (57%) showed an adenocarcinoma histology, while 27 (31%) exhibited a squamous cell carcinoma histology. PD‐L1 expression was ≥1% in 72 patients (83%) but was absent in 13 (15%). Twenty‐seven patients (31%) received ICI as first‐line therapy. Sixty patients (69%) received ICI as a second‐line or later treatment. Seven (8%) had liver metastasis. Twenty‐three patients (26%) had brain metastasis. Six patients (7%) received oral steroids regularly during the treatment.

**TABLE 2:** Kinetics of white blood cell counts over time according to the response type.

Total		Pre‐treatment	2 weeks later	4 weeks later	6 weeks later
		(*n* = 151)	(*n* = 138)	(*n* = 151)	(*n* = 143)
White blood count/μL	Mean, (SD)	6826 (2597)	7481 (3739)^a^	7929 (5170)^b^	7690 (5478)^a^
Neutrophils/μL	Mean, (SD)	4647 (2231)	5233 (3464)	5625 (4993)^a^	5347 (5282)
Neutrophils, %	Mean, (SD)	66 (9.3)	67 (11)	67 (12)^b^	66 (13)
Lymphocytes/μL	Mean, (SD)	1440 (581)	1456 (615)	1516 (694)	1498 (667)
Lymphocytes, %	Mean, (SD)	22 (8.7)	22 (9.5)	22 (10)	23 (11)
Eosinophils/μL	Mean, (SD)	179 (187)	216 (210)^b^	218 (246)^a^	209 (265)
Eosinophils, %	Mean, (SD)	2.7 (2.5)	3.3 (3.1)^b^	3.3 (3.4)^a^	3.3 (3.6)
sNLR, ratio	Mean, (SD)	3.6 (2.0)	4.3 (3.6)^b^	5.2 (7.5)^b^	5.5 (10.6)^a^

Responders		(n = 50)	(n = 45)	(n = 50)	(n = 50)
White blood count/μL	Mean, (SD)	6656 (2548)	6590 (2979)	6302 (2483)	6212 (2154)
Neutrophils/μL	Mean, (SD)	4521 (2242)	4496 (2796)	3938 (2085)	3753 (1586)^a^
Neutrophils, %	Mean, (SD)	66 (10)	64 (12)	61 (10)^b^	59 (11)^b^
Lymphocytes/μL	Mean, (SD)	1426 (519)	1447 (592)	1620 (618)^a^	1686 (695)^b^
Lymphocytes, %	Mean, (SD)	23 (9.0)	24 (11)	27 (8.8)^b^	28 (9.6)^b^
Eosinophils/μL	Mean, (SD)	144 (94)	175 (138)^a^	190 (118)^b^	224 (318)
Eosinophils, %	Mean, (SD)	2.5 (1.8)	3.1 (2.8)	3.3 (2.1)^b^	3.5 (2.9)^a^
sNLR, ratio	Mean, (SD)	3.6 (2.1)	3.8 (3.5)	2.7 (1.6)^b^	2.6 (2.0)^b^

Nonresponders		(*n* = 101)	(*n* = 93)	(*n* = 101)	(*n* = 93)
White blood count/μL	Mean, (SD)	6911 (2630)	7922 (4005)^a^	8735 (5924)^b^	8475 (6474)^a^
Neutrophils/μL	Mean, (SD)	4709 (2234)	5590 (3706)^a^	6459 (5757)^b^	6203 (6293)^a^
Neutrophils, %	Mean, (SD)	67 (8.8)	68 (10)	69 (12)^b^	70 (13)^a^
Lymphocytes/μL	Mean, (SD)	1447 (612)	1460 (629)	1465 (726)	1397 (633)
Lymphocytes, %	Mean, (SD)	22 (8.5)	21 (8.7)^b^	20 (10)^a^	20 (10)^a^
Eosinophils/μL	Mean, (SD)	196 (217)	236 (235)^a^	232 (288)	202 (233)
Eosinophils, %	Mean, (SD)	2.9 (2.8)	3.4 (3.3)^a^	3.3 (3.8)	3.1 (3.9)
sNLR, ratio	Mean, (SD)	3.7 (2.0)	4.5 (3.7)^b^	6.4 (8.9)^b^	7.1 (12.9)^b^

*Note*: Comparisons with pretreatment values were performed with the paired *t*‐test.

Abbreviations: Responders, patients with a complete or partial response; nonresponders, patients with stable or progressive disease; sNLR, neutrophil‐to‐lymphocyte ratio in serum.

^a^ *p* <0.05.

^b^ *p* <0.01.

The mean age of 64 patients without an increased REC was 69 years; 50 (78%) were male, and 54 (84%) were ex‐ or current smokers. Fifty‐six patients (88%) had an ECOG PS of 0–1. Nine patients (14%) had postoperative recurrence, 11 (17%) had stage III, and 44 (69%) had stage IV disease. Thirty‐nine patients (61%) exhibited an adenocarcinoma histology, while 16 (25%) showed a squamous cell carcinoma histology. PD‐L1 expression was ≥1% in 46 patients (72%) but was absent in 17 (27%). Thirteen patients (20%) received ICIs as first‐line therapy. Fifty‐one patients (80%) received ICIs as a second‐line or later treatment. Six patients (9%) had liver metastasis. Eleven patients (17%) had brain metastasis. Three patients (5%) received oral steroids regularly during the treatment.

The baseline tumor size, white blood cell count, neutrophil count, lymphocyte count, sNLR, and CRP did not significantly differ between the two groups. However, significant differences were noted in the baseline eosinophil count and Alb. Furthermore, after 4 weeks of ICI treatment, significant differences were observed in white blood cell, neutrophil, relative lymphocyte, and eosinophil counts in the two groups.

### RR and DCR with and without an increased REC

**FIGURE 1:**
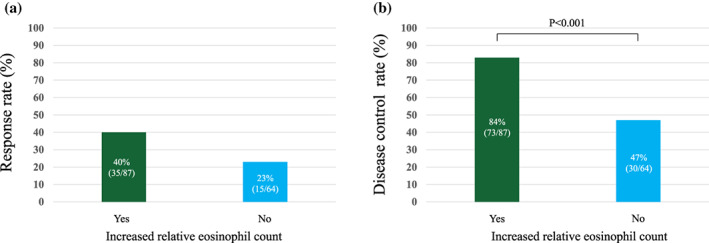
(a) Response rate and (b) disease control rate with and without an increased relative eosinophil count after 4 weeks of treatment with immune checkpoint inhibitors.

RR was higher in patients with than in those without an increased REC (40% [95% CI: 30–51%] vs. 23% [95% CI: 13–34%], Figure 1a). DCR was significantly higher in patients with than in those without an increased REC (84% [95% CI: 76–92%] vs. 47% [95% CI: 34–59%], *p* < 0.001; Figure 1b).

### OS of NSCLC patients treated with ICIs

The median OS of all 151 NSCLC patients treated with ICIs was 476 days (95% CI: 338–614).

### OS of NSCLC patients treated with ICIs according to an increased REC

**FIGURE 2:**
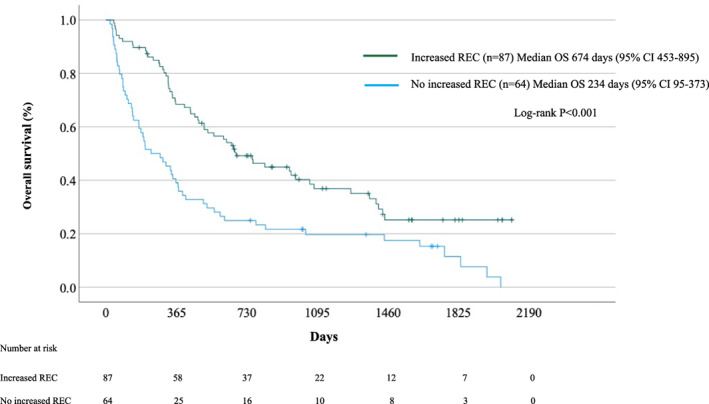
Survival of patients with and without an increased relative eosinophil count (REC) after 4 weeks of treatment with immune checkpoint inhibitors.

The median OS of NSCLC patients with an increased REC after 4 weeks of the initial ICI treatment (n = 87) and without an increased REC (*n* = 64) were 674 days (95% CI: 453–895) and 234 days (95% CI: 95–373), respectively (Figure 2). Median OS was significantly longer in NSCLC patients with than in those without an increased REC (*p* < 0.001).

#### Univariate analysis

As shown in Table 3, univariate Cox proportional regression analyses identified an ECOG PS score ≥2 (HR, 1.85; 95% CI: 1.02–3.40; *p* = 0.045), liver metastasis (HR, 1.95; 95% CI: 1.04–3.65; *p* = 0.04), 100 ≤ Eo < 500 (HR, 0.30; 95% CI: 0.14–0.66; *p* = 0.003), an increased REC (HR, 0.49; 95% CI: 0.34–0.71; *p* < 0.001), increased relative neutrophil count (RNC) (HR, 1.98; 95% CI: 1.07–3.65; *p* = 0.03), increased relative lymphocyte count (RLC) (HR, 0.54; 95% CI: 0.37–0.79; *p* = 0.001), CRP ≥1 mg/dL (HR, 1.49; 95% CI: 1.03–2.15; *p* = 0.04), Alb ≥3.5 g/dL (HR, 0.68; 95% CI: 0.47–0.999; *p* = 0.049), and tumor size ≥5 cm (HR, 1.81; 95% CI, 1.24–2.64; *p* = 0.002) as significant factors for OS during ICI monotherapy, but not age, sex, stage, histological type, brain metastasis, sNLR, or PD‐L1 ≥ 1%.

**TABLE 3:** Univariate Cox regression analysis of clinical and laboratory parameters associated with the overall survival of non‐small cell lung cancer patients treated with immune checkpoint inhibitors.

Parameters	Category	Hazard ratios	95% CI of HR	*p*‐value
ECOG PS	2–4	1.85	1.02–3.40	0.045
	0–1	Reference		
Age	≥75	0.97	0.62–1.50	0.9
	<75	Reference		
Sex	Female	1.09	0.87–1.35	0.5
	Male	Reference		
Stage	Recurrence	0.65	0.36–1.18	0.2
	III	0.72	0.44–1.16	0.2
	IV	Reference		
Histological type	Sq	1.15	0.75–1.66	0.6
	Non‐Sq	Reference		
Liver metastasis	Yes	1.95	1.04–3.65	0.04
	No	Reference		
Brain metastasis	Yes	1.34	0.87–2.05	0.20
	No	Reference		
Eosinophils	<100/μL	0.50	0.22–1.12	0.09
	≤100/μL, <500/μL	0.30	0.14–0.66	0.003
	≥500/μL	Reference		
Increased REC	Yes	0.49	0.34–0.71	<0.001
	Νο	Reference		
Increased RNC	Yes	1.98	1.07–3.65	0.03
	Νο	Reference		
Increased RLC	Yes	0.54	0.37–0.79	0.001
	Νο	Reference		
sNLR, ratio	<5	1.08	0.65–1.78	0.8
	≥5	Reference		
CRP	≥1 mg/dL	1.49	1.03–2.15	0.04
	<1 mg/dL	Reference		
Alb	≥3.5 g/dL	0.68	0.47–0.999	0.049
	<3.5 g/dL	Reference		
Tumor size	≥5 cm	1.81	1.24–2.64	0.002
	<5 cm	Reference		
PD‐L1	≥1%	0.67	0.43–1.03	0.07
	<1%	Reference		

Abbreviations: Alb, albumin; CI, confidence interval; CRP, C‐reactive protein; ECOG PS, Eastern Cooperative Oncology Group performance status; HR, hazard ratio; Non‐Sq, nonsquamous cell carcinoma; PD‐L1, programmed death ligand 1; REC, relative eosinophil count; RLC, relative lymphocyte count; RNC, relative neutrophil count; sNLR, neutrophil‐to‐lymphocyte ratio in serum; Sq, squamous cell carcinoma.

#### Multivariate analysis

Variables with *p*‐values ≤ 0.2 in univariate models were analyzed in multivariate models. In the Cox proportional regression analysis, brain metastasis (HR, 1.70; 95% CI: 1.04–2.76; *p* = 0.03), 100 ≤ Eo < 500 (HR, 0.26; 95% CI: 0.09–0.74; *p* = 0.01), an increased REC (HR, 0.52; 95% CI: 0.34–0.80; *p* = 0.003), increased RNC (HR, 3.32; 95% CI: 1.47–7.53; *p* = 0.004), tumor size ≥5 cm (HR, 2.19; 95% CI: 1.26–3.80; *p* = 0.006), and PD‐L1 ≥ 1% (HR, 0.58; 95% CI: 0.36–0.94; *p* = 0.03) correlated with OS (Table 4), whereas ECOG PS, stage, liver metastasis, an increased RLC, CRP, and Alb did not.

**TABLE 4:** Multivariate Cox regression analysis of clinical and laboratory parameters associated with the overall survival of non‐small cell lung cancer patients treated with immune checkpoint inhibitors.

Parameters	Category	Hazard ratios	95% CI of HR	*p*‐value
ECOG PS	2–4	1.97	0.96–4.05	0.07
	0–1	Reference		
Stage	Recurrence	0.69	0.37–1.29	0.2
	III	0.69	0.39–1.24	0.2
	IV	Reference		
Liver metastasis	Yes	0.92	0.44–1.93	0.8
	No	Reference		
Brain metastasis	Yes	1.70	1.04–2.76	0.03
	No	Reference		
Eosinophils	<100/μL	0.47	0.16–1.37	0.1
	≤100/μL, <500/μL	0.26	0.09–0.74	0.01
	≥500/μL	Reference		
Increased REC	Yes	0.52	0.34–0.80	0.003
	Νο	Reference		
Increased RNC	Yes	3.32	1.47–7.53	0.004
	Νο	Reference		
Increased RLC	Yes	0.86	0.54–1.35	0.5
	Νο	Reference		
CRP	≥1 mg/dL	1.27	0.73–2.19	0.4
	<1 mg/dL	Reference		
Alb	≥3.5 g/dL	1.14	0.66–1.96	0.6
	<3.5 g/dL	Reference		
Tumor size	≥5 cm	2.19	1.26–3.80	0.006
	<5 cm	Reference		
PD‐L1	≥1%	0.58	0.36–0.94	0.03
	<1%	Reference		

Abbreviations: Alb, albumin; CI, confidence interval; CRP, C‐reactive protein; ECOG PS, Eastern Cooperative Oncology Group performance status; HR, hazard ratio; PD‐L1, programmed death ligand 1; REC, relative eosinophil count; RLC, relative lymphocyte count; RNC, relative neutrophil count.

#### Validation

The multivariate Cox proportional regression analysis was validated using a bootstrap analysis with 1000 resamples processed internally. In the Cox proportional regression analysis, brain metastasis (HR, 1.70; 95% CI: 0.89–3.23; *p* = 0.07), 100 ≤ Eo < 500 (HR, 0.26; 95% CI: 0.07–1.11; *p* = 0.02), an increased REC (HR, 0.52; 95% CI: 0.29–0.82; *p* = 0.006), increased RNC (HR, 3.32; 95% CI: 1.25–10.3; *p* = 0.02), tumor size ≥5 cm (HR, 2.19; 95% CI: 1.04–4.83; *p* = 0.02), and PD‐L1 ≥ 1% (HR, 0.58; 95% CI: 0.31–1.03; *p* = 0.045) correlated with OS. These results confirmed validity.

## DISCUSSION

The present results demonstrated that ICI‐treated NSCLC patients with an increased REC after 4 weeks of treatment had a better DCR and prognosis than the other patients examined. To the best of our knowledge, this is the first multivariate study to show that an increased REC after 4 weeks of treatment is a significant predictive factor of lung cancer patients treated with ICIs.

Eosinophils are effector cells in allergic diseases and parasitic infections. However, eosinophils were recently shown to function as multifaceted leukocytes that contribute to various physiological and pathological processes depending on their location and activation state.^5^, ^6^ Furthermore, eosinophils are considered to regulate homeostatic processes at a steady state.^7^ Previous studies demonstrated that tumor‐related eosinophilia may prolong the survival of some cancer patients.^8^, ^9^, ^10^ We also showed that the prognosis of lung cancer patients with eosinophilic pleural effusion was better than that of patients with noneosinophilic effusion.^11^ Similar findings have been reported for many cancers, including lung cancer.^24^, ^25^, ^26^, ^27^, ^28^, ^29^, ^30^ Meta‐analyses also revealed that tumor‐associated tissue eosinophils predicted favorable clinical outcomes in solid tumors.^13^ Furthermore, immunotherapy with interleukin (IL)‐2,^31^, ^32^ IL‐4,^33^ granulocyte‐macrophage colony‐stimulating factor,^34^ or tumor vaccines often results in peripheral eosinophilia.^35^ The intrapleural administration of IL‐2 was previously reported to induce significant eosinophilic pleural effusion.^36^ Conversely, in Hodgkin's lymphoma, infiltrating eosinophils into tumor‐associated tissues is regarded as a poor prognostic factor.^37^ Eosinophils have been shown to play pleiotropic and opposing roles in the tumor microenvironment.^38^, ^39^, ^40^

In urothelial carcinoma patients treated with pembrolizumab, an increased REC at 3 weeks has already been identified as a significant predictive factor.^14^ A maximum eosinophil count of 5% during ICI therapy is used as an essential predictor of the time to treatment failure in lung cancer patients.^15^, ^17^ In NSCLC patients, ICI therapy only induces an early increase in blood eosinophils, which is more prominent in responding patients.^16^ An increased REC during ICI therapy correlated with more objective responses.^16^ Furthermore, an increased REC during ICI therapy was associated with a longer duration of ICI therapy in lung cancer patients.^16^ Clinical data suggest that an increased REC reflects a favorable outcome in ICI‐treated NSCLC patients.^19^ However, OS is the most robust parameter for the outcome of cancer treatment. The relationship between an increased REC and the prognosis of lung cancer patients treated with ICIs has not yet been reported in detail.^19^ In the present study, the multivariable analysis identified brain metastasis (*p* = 0.03), 100 ≤ Eo < 500 (*p* = 0.01), an increased REC (*p* = 0.003), increased RNC (*p* = 0.004), tumor size ≥5 cm (*p* = 0.006), and PD‐L1 ≥ 1% (*p* = 0.03) as independent predictors of OS. Except for an increased REC, these results are consistent with previous findings.^1^, ^12^, ^22^, ^23^ In previous studies, the median time to the maximum eosinophil percentage was 5 weeks in lung cancer patients with controlled disease and 2 weeks in those with advanced disease.^15^ Under normal conditions, eosinophil production is tightly regulated by the cytokine network.^41^ An increased REC following 4 weeks of treatment may be an essential early predictive biomarker of improved clinical outcomes for ICI‐treated NSCLC patients. Similarly, an increased absolute eosinophil count after 4 weeks of treatment significantly affected the prognosis of patients. The results were the same even when the nine patients regularly using oral steroids were excluded. Eosinophil levels and changes in NLR dynamics predicted clinical outcomes more accurately than the tumor mutation burden and PD‐L1 expression.^42^ The early dynamics of peripheral blood immune cell subsets may reflect changes in the tumor microenvironment, capture antitumor immune responses, and ultimately reflect clinical outcomes with ICIs.^42^ Therefore, an increased REC following 4 weeks of treatment may be valuable as a dynamic biomarker in lung cancer patients treated with ICIs.

Eosinophils are directly cytotoxic to cancer cells through degranulation.^43^, ^44^ They polarize macrophages to an antitumor (M1) phenotype.^45^ Activated eosinophils recruit, activate, and induce the maturation of several immune cells, such as natural killer cells, T cells, and dendritic cells, and also promote tumor rejection.^39^, ^43^, ^45^, ^46^, ^47^ In addition, eosinophils have been suggested to normalize tumor vessels.^45^ Eosinophils activated by ICIs may facilitate the migration of CD8^+^ T cells to the tumor site in melanoma patients.^48^ In colorectal cancer models, tumor‐infiltrating eosinophils have been reported to consist of degranulating eosinophils and are essential for tumor rejection independently of CD8+ T cells.^49^ Furthermore, eosinophils have been suggested to contribute to plasma B cell survival through a proliferation‐inducing ligand and IL‐6.^50^ In clinical practice, IL‐2 leads to an increase in IL‐5, a crucial growth, differentiation, and activating factor for eosinophils, which results in eosinophilia.^51^ An increased REC may play an essential role in cancer immunology and immunotherapy.

The results of this study need to be interpreted in consideration of some limitations. External validity was not examined due to the small sample size. This study was a two‐center retrospective analysis conducted with heterogeneous data from patient cohorts, and, as such, the results obtained are speculative and not definitive. Therefore, a long‐term follow‐up in a large prospective study is needed to validate the present results. We demonstrated the importance of pretreatment eosinophil counts and early mortality factors in ICI monotherapy for advanced or metastatic NSCLC.^12^, ^21^ The same population was examined as that in our previous study. However, previous research identified early mortality factors and the importance of pretreatment eosinophil counts during ICI treatment. The present study focused on the impact of an increased REC on the effectiveness of ICIs for NSCLC and the long‐term prognosis of treated patients. These studies focused on entirely different and essential issues. Although we need to consider these limitations when interpreting the present results, this study is valuable because an increased REC was confirmed for the first time to be a favorable predictive factor using a multivariate analysis of ICI monotherapy for advanced and metastatic NSCLC. An increased REC 4 weeks after the initial administration of ICIs was associated with longer OS. Moreover, an increased REC after 4 weeks of ICI treatment may be a valuable and inexpensive dynamic predictive biomarker in clinical practice for early assessments of ICIs. The role of eosinophils in cancer immunology and immunotherapy has not yet been elucidated in detail. However, they may be essential accessory cells for cancer immunology and immunotherapy. A more detailed understanding of the relationship between eosinophils, cancer immunology, and cancer immunotherapy is warranted.

In conclusion, ICI‐treated NSCLC patients with an increased REC after 4 weeks of treatment had a better DCR and prognosis than the other patients examined and thus an increased REC has potential as an early predictive dynamic biomarker.

## AUTHOR CONTRIBUTIONS

Conceptualization and design: E. T. and H. N.; Data collection and analysis: H. O., K. K., Y. O., S. I., M. K., N. K., H. M., N. H., K. N., and T. S.; Formal analysis and investigation: E. T.; Writing—original draft preparation: E. T.; Writing—review and editing: H. O., H. N., T. S., and Y. N.; Supervision: H. N., T. S., and Y. N. All authors read and approved the final manuscript.

## CONFLICT OF INTEREST STATEMENT

The authors declare that they have no conflicts of interest.
